# Effects of T5 Treatment on Microstructure and Mechanical Properties at Elevated Temperature of AZ80-Ag Alloy

**DOI:** 10.3390/ma12193214

**Published:** 2019-09-30

**Authors:** Gang Zeng, Chuming Liu, Yonghao Gao, Shunong Jiang, Shilun Yu, Zhiyong Chen

**Affiliations:** 1 School of Materials Science and Engineering, Central South University, Changsha 410083, China; 2 School of Materials Science and Engineering, Hunan University of Science and Technology, Xiangtan 411201, China

**Keywords:** AZ80-Ag alloy, T5 treatment, precipitation, creep

## Abstract

Effects of T5 treatment on microstructure and mechanical properties at elevated temperature of hot-ring-rolled (HRRed) AZ80-Ag magnesium alloy were systematically investigated. Results show that, after aging at 175 °C for 36 h, discontinuous and continuous precipitates form inside grains, with the former one taking up a volume fraction of ~64.9%. T5 treatment improves the tensile strength at ambient temperature of the alloy but weakens its tensile strength and creep resistance at elevated temperatures (120–175 °C), indicating opposite effects of T5 on mechanical properties at ambient and elevated temperatures. During creep at 120–175 °C and under 70–90 MPa, the dynamic precipitation process in HRRed specimen is accelerated with increasing temperature. At 150–175 °C massive nucleation and growth of dynamic discontinuous precipitates could result in an atypical primary creep stage, consisting of deceleration and acceleration creep stages, which is reported in wrought Mg-Al-based alloy for the first time. Such primary creep stage can be eliminated by T5 treatment. Besides, diffusion-controlled dislocation creep is the dominant creep mechanism for both HRRed and T5 specimens.

## 1. Introduction

Magnesium alloys are promising engineering materials due to their low density, high specific strength, and superior electromagnetic shielding performance [1,2,3]. Compared with cast magnesium alloys, wrought alloys generally exhibit higher strength and better plasticity, and are suitable for load-bearing parts. Among various magnesium alloys, AZ80 alloy can be widely utilized owing to its desirable combination of low cost, moderate strength and reasonable ductility [4]. To further strengthen the alloy, micro-alloying is believed to be an effective method. According to our previous studies [5,6,7,8], Ag is an effective alloying element for AZ80 alloy, as demonstrated by the enhanced age-hardening response and excellent plastic formability of Ag-containing alloys. Therefore, wrought AZ80-Ag alloy has broad application prospect in automotive, aviation and aerospace areas.

In practical applications, aging treatment is generally conducted to modify comprehensive performance of wrought AZ80-based alloys [5,9,10,11,12]. Previous studies are mainly focused on the effect of heat treatment on mechanical properties at ambient temperature, reporting the strengthening effect through the formation of β-Mg_17_Al_12_ phase. However, studies about the effect of aging treatment on mechanical properties at elevated temperature are rarely reported. As load-bearing parts in aircraft or spacecraft, magnesium alloy components need to work at elevated temperature in some cases, rendering the components subjected to thermal-mechanical coupled loads. Therefore, creep performance is an important parameter to evaluate the long-term reliability.

Extensive researches have been performed on the creep behavior of cast Mg-Al-based alloys [13,14,15,16]. It is revealed that dynamic precipitation of discontinuous and continuous Mg_17_Al_12_ phase is a major microstructural evolution during creep, which in turn has significant influence on creep behavior. Discontinuous precipitation is believed to have a detrimental effect on creep resistance due to promotion of grain boundary sliding [15,16]. In contrast, continuous precipitation exhibits a strengthening effect at relatively low temperatures [16]. Up to now, little information can be obtained about the role of dynamic precipitation in creep of wrought alloys.

In a recent study by Zeng et al. [17], aging temperature in T6 treatment was shown to significantly influence the microstructure and creep performance of AZ80-Ag alloy. However, in that study nothing was discussed about the comparison of mechanical properties at elevated temperature between wrought and aged alloys, or the influence of dynamic precipitation on creep behavior. In this study, we investigated the effects of aging treatment on mechanical properties at elevated temperature of wrought AZ80-Ag alloy and the dynamic precipitation during creep. We found that aging treatment improved tensile strength at ambient temperature but deteriorated tensile strength and creep resistance at elevated temperatures, and wrought AZ80-Ag alloy exhibited an atypical primary creep stage at 150–175 °C, which was eliminated by aging treatment.

## 2. Materials and Methods

In the present study, a hot-ring-rolled (HRRed) AZ80-Ag alloy (8.10 Al, 0.46 Zn, 0.18 Mn, 0.18 Ag, balance Mg, wt%) was used, and its fabrication process has been depicted in our previous work [7]. Specimens used in this study were machined from the region near outer surface of the ring. Previous result indicates that the alloy can reach a peak hardness after aging at 175 °C for 36 h [5,6]. Thus, a group of HRRed specimens were peak-aged at 175 °C for 36 h, denoted as T5 specimens.

Microstructures were characterized using Leica optical microscope (OM, Wetzlar, Germany), FEI Nova Nano230 scanning electron microscope (SEM, Hillsboro, OR, USA) and FEI Tecnai G20 transmission electron microscope (TEM, Hillsboro, OR, USA). OM and SEM samples were prepared by mechanical polishing and etching. TEM samples were prepared by argon ion-beam thinning. Macro-texture was identified using Bruker D8 Discover X-ray diffractometer (Billerica, MA, USA). OM, SEM and texture examinations were conducted in the rolling plane, i.e., rolling-axial direction (RD-AD) plane [7]. Volume fraction of precipitation was determined from three SEM images using Image-Pro Plus 6.0 software (6.0.0.260, Media Cybernetics, Rockville, MD, USA).

Tensile tests were carried out at ambient (25 °C) and elevated temperatures (120 °C and 175 °C) using Instron 3369 testing machine (Instron, Boston, MA, USA) with a cross-head speed of 1 mm/min. The tensile specimens with a gauge dimension of Φ4 mm × 20 mm were machined from the ring along AD. To ensure the reliability of the data, three tensile tests were repeated for each condition and their average results were reported. Tensile creep tests were performed using CRITM RWS50 creep testing machine (CRITM, Changchun, China), at temperatures of 120–175 °C and under constant loads of 70–90 MPa. The creep specimens, with a gauge dimension of Φ10 mm × 50 mm, were cut from the ring along AD. Specimens were crept up to 100 h unless they were fractured within that time. Before loading, the specimens were preheated to target temperature and held for 1 h. The creep displacement was measured using two linear variable differential transformers (LVDT) attached to the high-temperature extensometer, coupled with a data acquisition system.

## 3. Results

### 3.1. Microstructure and Texture Evolution after T5 Treatment

**Figure 1:**
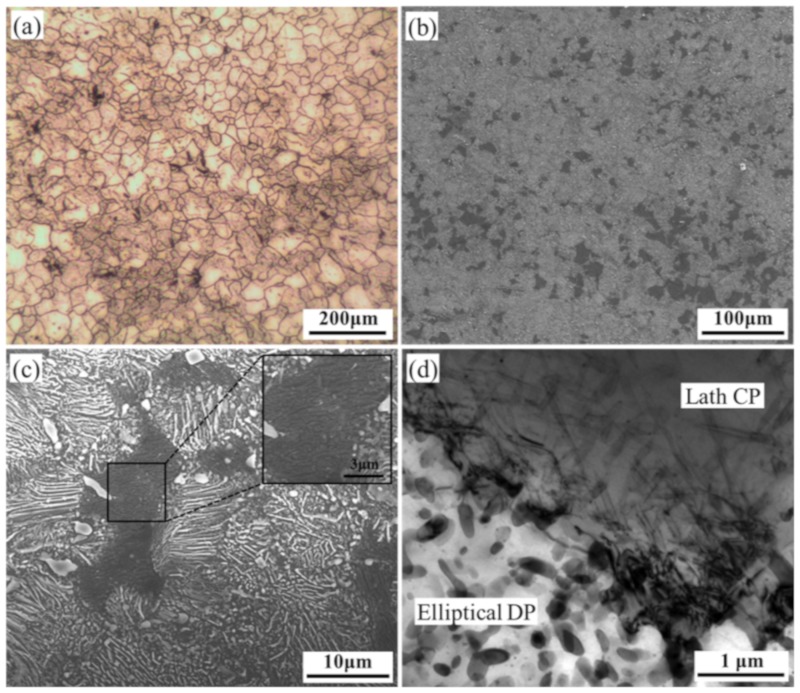
Microstructure of (**a**) hot-ring-rolled (HRRed) and (**b**–**d**) T5 specimens.

Microstructure of HRRed specimen is presented in Figure 1a. The examined region is mainly comprised of recrystallized grains, with an average size of ~40 μm. After T5 treatment, numerous β-Mg_17_Al_12_ precipitates are formed, as shown in Figure 1b–d. According to their morphologies, β-Mg_17_Al_12_ precipitates in Mg-Al series alloys are classified into two types, continuous precipitation (CP) and discontinuous precipitation (DP) [18]. In T5 specimen, most grains are occupied by bright lamellar and elliptical β precipitates, known as typical DP [18]. Meanwhile, a small area fraction of dark region can be found in Figure 1b. The magnified image embedded in Figure 1c reveals the presence of fine lath-shaped precipitates, i.e., typical CP [18]. Statistical analysis indicates that the volume fraction of DP is about 64.9%. It is verified in TEM image (Figure 1d) that DP and CP occupy different regions with obvious boundary between them. Due to relatively low stored energy and aging temperature (175 °C), little change in grain size would occur during T5 treatment.

**Figure 2:**
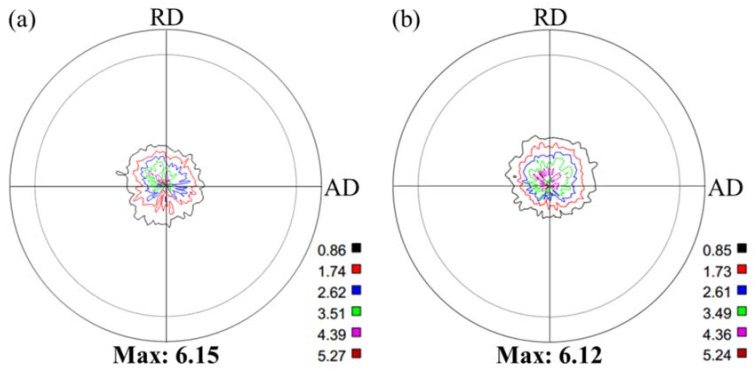
(0002) pole figures of (**a**) HRRed and (**b**) T5 specimens.

In order to capture the texture evolution during T5 treatment, (0002) pole figures are displayed in Figure 2. A typical basal texture is formed during HRR process (Figure 2a), indicating that most grains are oriented with their {0002} planes parallel to the rolling plane. It is noted that the basal texture is retained after T5 treatment (Figure 2b). Furthermore, except for a slight change in maximum pole value, little distinction is revealed between the pole figures of HRRed and T5 specimens.

### 3.2. Tensile Properties at Ambient and Elevated Temperatures

**Figure 3:**
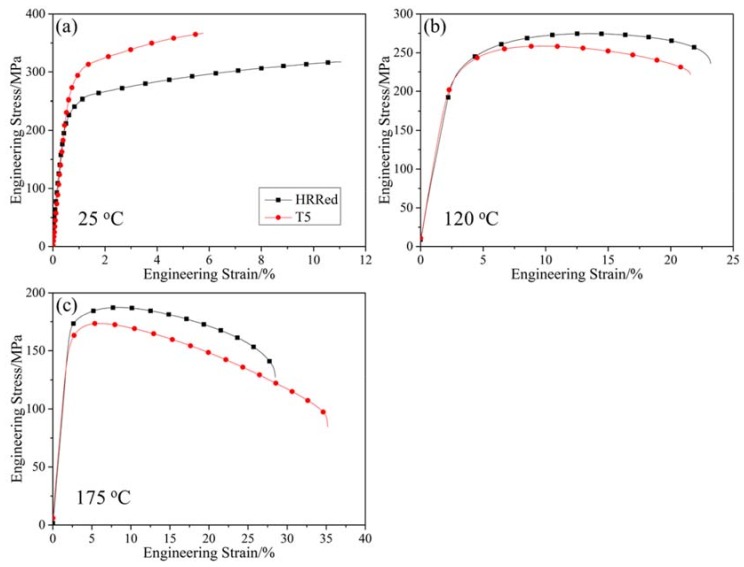
Tensile curves of HRRed and T5 specimens tested at different temperatures. (**a**) 25 °C; (**b**) 120 °C; (**c**) 175 °C.

Tensile curves of specimens tested at ambient and elevated temperatures are shown in Figure 3. Corresponding tensile properties are presented in Table 1, including the values of yield strength (YS), ultimate tensile strength (UTS) and elongation to failure. It is found that the tensile strength at ambient temperature of AZ80-Ag alloy is evidently enhanced by T5 treatment, with a 17.7% increase in YS and a 14.9% increase in UTS. However, at temperatures over 120 °C, T5 specimen exhibits lower YS and UTS than HRRed specimen. Thus, T5 treatment has opposite effects on tensile properties at ambient and elevated temperatures.

### 3.3. Creep Performance and Dynamic Microstructural Evolution

**Figure 4:**
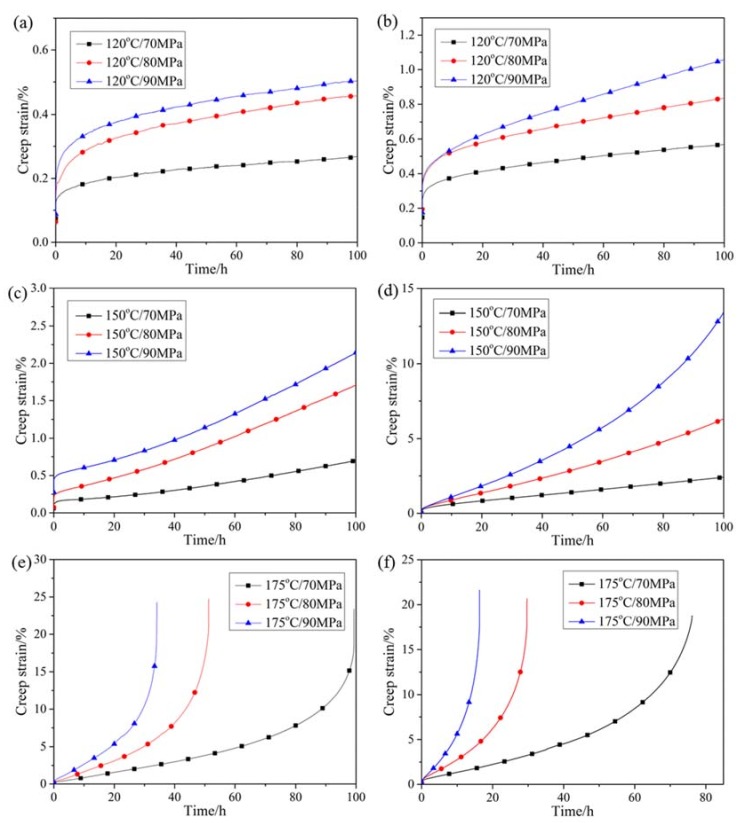
Creep curves of (**a**,**c**,**e**) HRRed and (**b**,**d**,**f**) T5 specimens tested at 120–175 °C and under 70–90 MPa.

**Figure 5:**
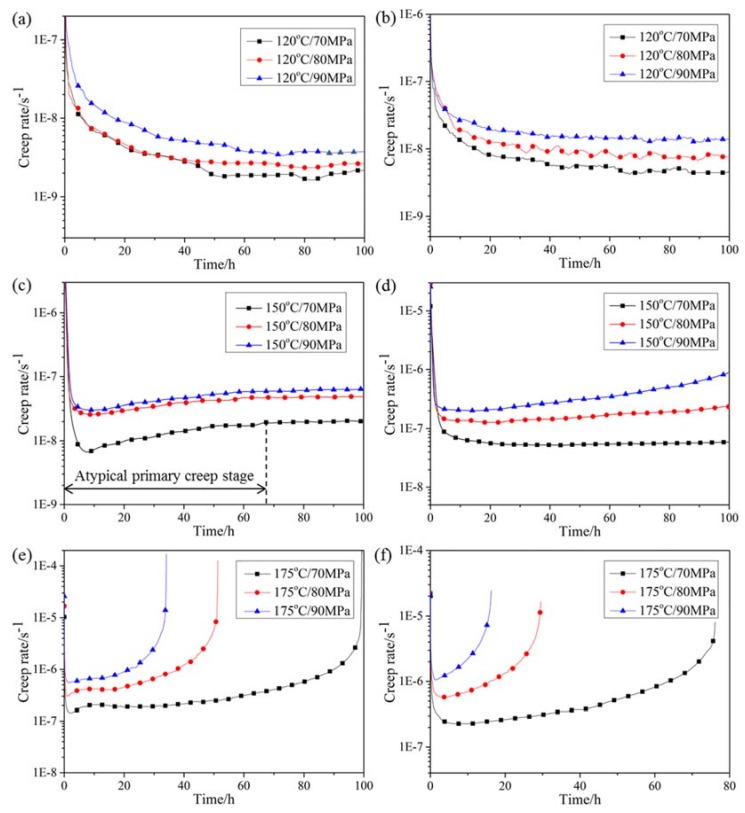
Creep rate versus time plots of (**a**,**c**,**e**) HRRed and (**b**,**d**,**f**) T5 specimens tested at 120–175 °C and under 70–90 MPa.

Creep curves of specimens tested at 120–175 °C and under 70–90 MPa are displayed in Figure 4. To illustrate the evolution of creep stage, corresponding creep rates are exhibited in Figure 5 as a function of testing time. As for HRRed specimens, the creep curves at 120 °C are comprised of deceleration creep stage and steady creep stage within 100 h. At temperatures over 150 °C, atypical primary creep stage, consisting of deceleration and acceleration creep stages, is revealed before steady creep stage, as illustrated in Figure 5c. Such primary creep stage is different from typical one characterized by continuously decelerating creep rate [19]. At 175 °C, secondary acceleration creep stage emerges, resulting in final fracture. As for T5 specimens, the creep curves at 120 °C are similar to those of HRRed specimens. With increasing temperature, typical creep curves are presented including primary deceleration creep stage, steady creep stage and acceleration creep stage.

Detailed creep data of HRRed and T5 specimens are summarized in Table 2 and Table 3, respectively. It is noted that T5 specimens show higher steady creep rate than HRRed specimens under the same condition, suggesting a detrimental effect of T5 treatment on creep performance.

**Figure 6:**
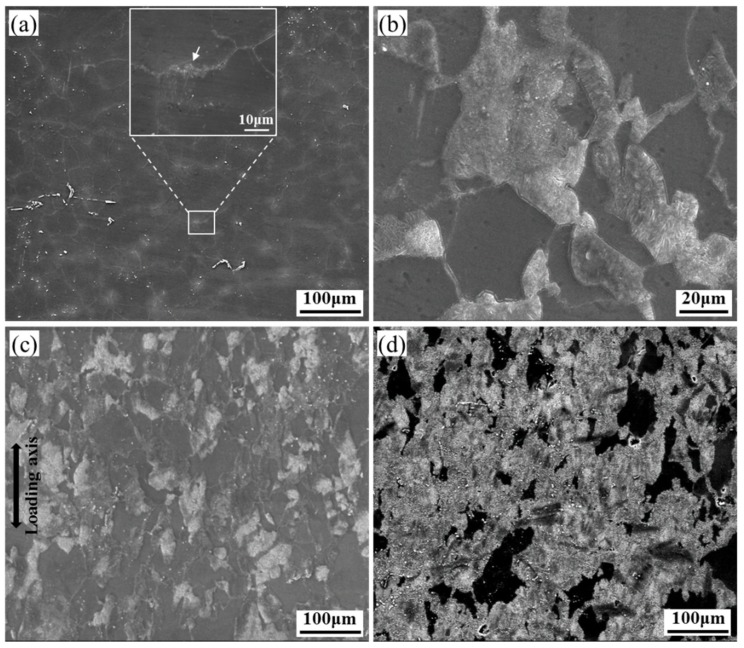
SEM images of HRRed specimens crept at (**a**) 120 °C/80 MPa to 100 h, 150 °C/80 MPa to (**b**) 35 h and (**c**) 100 h, and (**d**) 175 °C/80 MPa to fracture.

Figure 6 presents SEM micrographs of HRRed specimens crept under different conditions. For the specimen crept at 120 °C/80 MPa to 100 h, few dynamic precipitates are observed along grain boundaries, as marked by white arrow in Figure 6a. To analyze the abnormal acceleration creep stage, microstructure of the specimen crept at 150 °C/80 MPa to 35 h is shown in Figure 6b. With temperature increasing to 150 °C, the diffusion rate of solute atoms significantly increases, and therefore dynamic precipitation process is accelerated. The bright lamellar precipitates are mainly distributed in the vicinity of grain boundaries, indicating massive nucleation and growth of DP during primary creep stage. DP grows towards grain interior with creep time, and its volume fraction increases to ~58.8% after 100 h (Figure 6c). After crept at 175 °C/80 MPa to fracture (Figure 6d), the volume fraction of DP further increases to ~62.2%.

**Figure 7:**
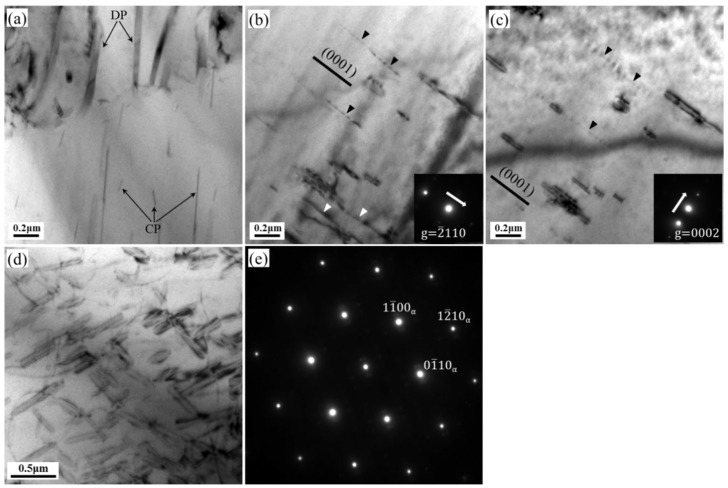
TEM images of HRRed specimens crept at (**a**–**c**) 150 °C/80 MPa and (**d**,**e**) 150 °C/90 MPa to 100 h. (**b**,**c**) are observed under two-beam conditions of *g* = $\bar{2}$ 110 and *g* = 0002 respectively. (**e**) is the diffraction pattern (B∥[0001]α) for (**d**). The traces of basal planes are indicated by black lines.

Figure 7 displays TEM images of HRRed specimens crept at 150 °C under 80–90 MPa to 100 h. Figure 7a reveals that fine continuous precipitates are formed in the region not occupied by discontinuous precipitates. Figure 7b and c are taken in the same area but under different two-beam diffraction conditions. It can be found that β precipitates are lying on the basal planes of the matrix, indicating that the applied stress does not alter common habit plane of β phase [20]. According to the visibility criterion of dislocation (*g* · *b* ≠ 0) [21], all the <a> type dislocations disappear when *g* = 0002, while all the <c> type dislocations are extinguished when *g* = $\bar{2}$ 110. However, the <c + a> dislocations do not lose the contrast under both conditions. In this sense, pure <a> dislocations, highlighted by white triangles, are revealed in Figure 7b. Besides, a few straight dislocation lines, marked by black triangles, are parallel to the trace of basal planes in Figure 7b,c, thus they are identified to be <c + a> dislocations. Dislocations of <c> type are rarely seen in this microstructure. In Figure 7d, numerous continuous precipitates, with lath-shaped and asymmetric lozenge-shaped morphologies, are observed on the basal plane, as verified by the diffraction pattern (Figure 7e). Moreover, these precipitates can act as obstacles to dislocation motion, resulting in dislocation pile-up and tangling.

**Figure 8:**
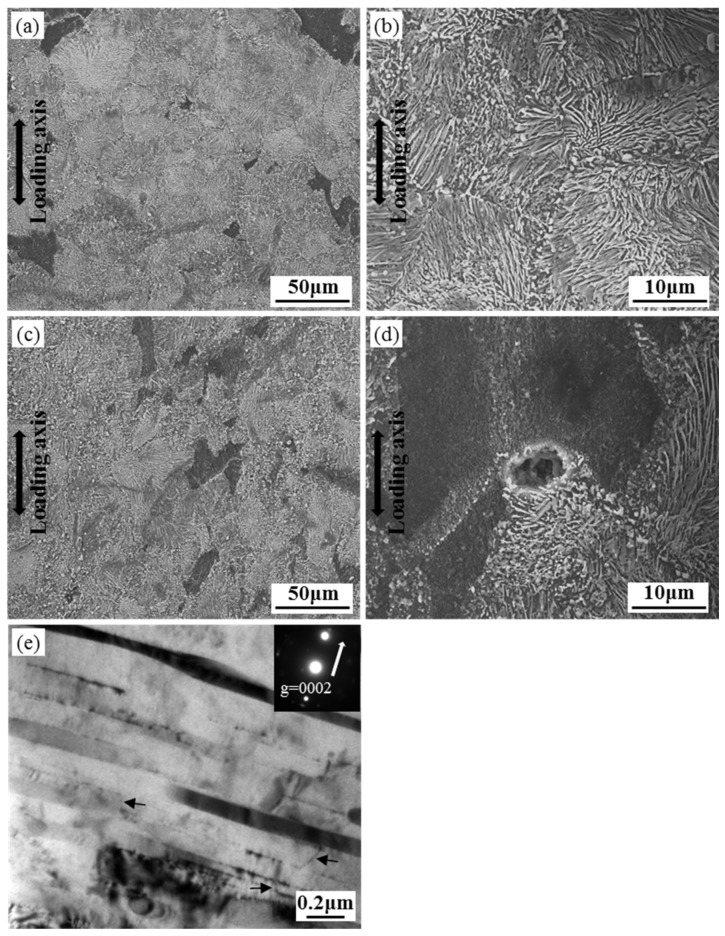
Microstructure of T5 specimens crept at (**a**,**b**) 150 °C/80 MPa and (**c**–**e**) 150 °C/90 MPa to 100 h.

Figure 8 shows the microstructure of T5 specimens crept at 150 °C under 80–90 MPa to 100 h. Under 80 MPa (Figure 8a,b), no obvious microstructural change is observed during steady creep stage, except for the coarsening of β precipitates. Comparing Figure 8b with Figure 1c reveals that lamellar precipitates are thickened and the lamellar spacing is narrowed. Under 90 MPa (Figure 8c–e), acceleration creep stage is presented. Apart from coarsening of precipitates, micro-crack is found along the interface between DP and CP. Under the two-beam condition of *g* = 0002 (Figure 8e), numerous dislocations with <c> component are observed. Some of these dislocations have kinks as marked by black arrows, which is probably associated with increasing dislocation density [21]. As creep strain increases dramatically during acceleration creep stage, the density and interaction of dislocations increase, resulting in more dislocations with kinked segments.

## 4. Discussion

### 4.1. Effects of T5 Treatment on Tensile Properties at Different Temperatures

The YS and UTS at ambient temperature of AZ80-Ag alloy are obviously enhanced by T5 treatment (Table 1). Generally, macro-texture and second phase are two controlling factors of tensile properties. Since little texture change is revealed after T5 treatment (Figure 2), the enhancement of tensile strength is mainly attributed to formation of β precipitates. These precipitates act as strong obstacles to dislocation motion and twinning at ambient temperature [22], leading to prominent precipitation hardening and, thus, higher values of YS and UTS.

**Figure 9:**
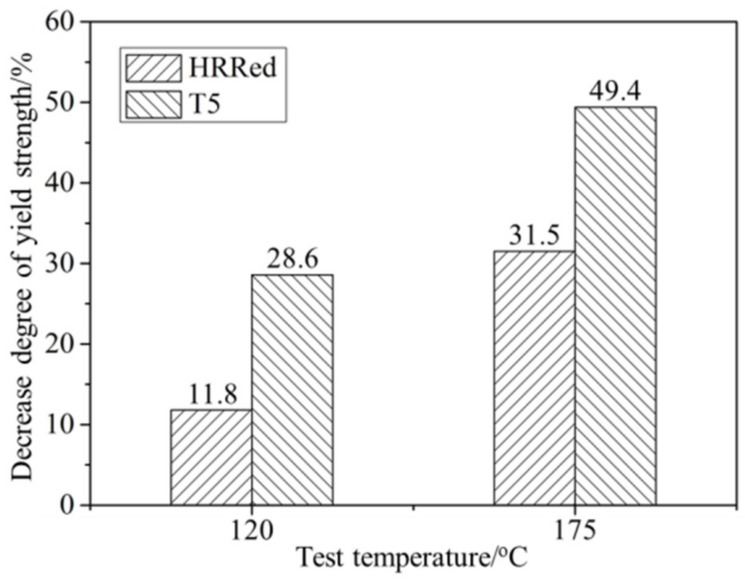
The relationship between decrease degree of yield strength and test temperature.

On the contrary, the YS and UTS at elevated temperatures of the alloy exhibit a downward trend after T5 treatment (Table 1). Since β-Mg_17_Al_12_ phase is prone to soften at temperatures over 120 °C due to its relatively low melting point (~437 °C) [19], such tensile strength deterioration may be related to the weakening of precipitation hardening effect. By contrast, most Al atoms are kept as solute atoms in HRRed specimen, providing solid solution strengthening against dislocation motion. As shown in Table 1, tensile strength of HRRed and T5 specimens decreases with increasing temperature. The decrease degree of YS ($\frac{{\mathrm{YS}}_{\mathrm{ambient}}-{\mathrm{YS}}_{\mathrm{elevated}}}{{\mathrm{YS}}_{\mathrm{ambient}}}$) is exhibited in Figure 9 as a function of test temperature. Apparently, T5 specimen exhibits higher decrease degree than HRRed specimen at both 120 °C and 175 °C. It is indicated that, compared with solid solution hardening, precipitation hardening is significantly weakened at 120–175 °C. Therefore, a detrimental effect of T5 on tensile strength of AZ80-Ag alloy is revealed at elevated temperatures.

### 4.2. Creep Mechanism Analysis

Applied stress (σ) and temperature (*T*) have significant influence on the steady creep rate ($\dot{\varepsilon }$) of magnesium alloys, and the relationship among them can be expressed by a power-law equation as follows [13,23,24,25]:

(1) $$\dot{\varepsilon }=A\sigma^{n}\exp(-Q/RT)$$

where *A* is material constant, *R* is gas constant, *n* is nominal creep stress exponent, and *Q* is creep activation energy. Particularly, *n* and *Q* are critical parameters clarifying creep mechanism. Equation (1) is then converted to Equation (2) as follows:

(2) $$\mathrm{Ln}\dot{\varepsilon }=\mathrm{Ln}A+n\mathrm{Ln}\sigma -Q/RT$$

At a given temperature, the *n* value can be obtained by Equation (3):

(3) $$n=\frac{\partial \ln\dot{\varepsilon }}{\partial \ln\sigma }|T$$

At a given stress, the *Q* value can be obtained by Equation (4):

(4) $$Q=-R\frac{\partial \ln\dot{\varepsilon }}{\partial \left(1/T\right)}|\sigma$$

**Figure 10:**
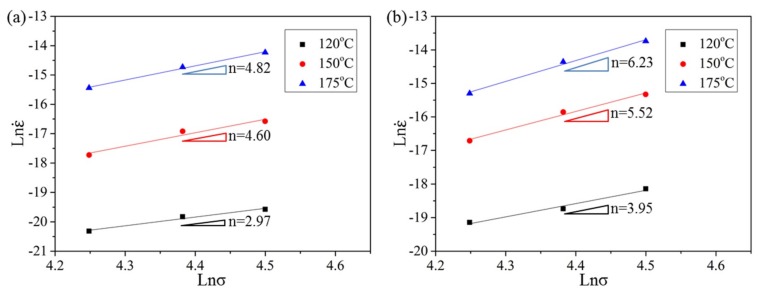
Stress dependence of the steady creep rate at 120–175 °C for (**a**) HRRed and (**b**) T5 specimens.

**Figure 11:**
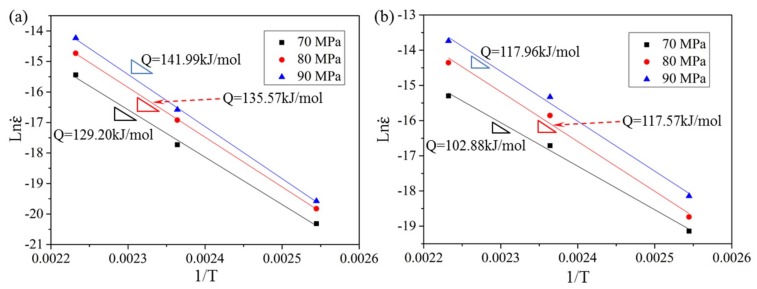
Temperature dependence of the steady creep rate under 70–90 MPa for (**a**) HRRed and (**b**) T5 specimens.

To determine the dominant creep mechanism of HRRed and T5 specimens, the *n* values are calculated by plotting ln $\dot{\varepsilon }$ against ln *σ* at 120–175 °C, as shown in Figure 10, and the *Q* values are calculated by plotting ln $\dot{\varepsilon }$ against 1/*T* under 70–90 MPa, as shown in Figure 11.

The *n* values of HRRed specimens are 2.97, 4.60, and 4.82 at 120 °C, 150 °C, and 175 °C respectively, while those of T5 specimens are 3.95, 5.52, and 6.23 at corresponding temperatures. For magnesium alloys, three types of creep mechanisms can be activated during creep deformation, grain boundary sliding (GBS), dislocation creep and diffusional creep, which are closely associated with the *n* value. Generally, *n* = 3–7 is reported for dislocation creep including dislocation viscous glide (*n* = ~3) and dislocation climb (*n* = 4–7) [25,26,27,28]. Therefore, the dominant creep mechanism is dislocation viscous glide at 120 °C, and dislocation climb at 150–175 °C for HRRed specimen. At low temperature, most Al atoms are in solid solution during creep process (Figure 6a), which can diffuse along dislocation pipes and exert drag force of dislocations. Consequently, the creep behavior can be interpreted in terms of viscous glide, in which dislocation movement is impeded by the solute atmosphere [26]. With temperature exceeding 150 °C, concentration of Al atoms in the matrix is remarkably reduced due to dynamic precipitation. Dislocation glide is inhibited by dynamic precipitates and dislocation pile-up occurs (Figure 7), thus dislocation climb can be activated for creep deformation. Accordingly, a transition from dislocation viscous glide creep to dislocation climb creep occurs, indicating important influence of dynamic precipitation on creep behavior. By contrast, the creep mechanism is always dislocation climb at 120–175 °C for T5 specimen. Consequently, dislocation motion is responsible for the creep of both specimens, consistent with TEM observations (Figure 7 and Figure 8e).

The information about specific diffusion mode in creep mechanism is revealed by *Q* value. For HRRed specimen, the *Q* values under 70–90 MPa are 129.20–141.99 kJ/mol, close to the activation energy for lattice self-diffusion of magnesium (135 kJ/mol) [15,29]. Therefore, lattice diffusion is determined to be the creep controlling factor. For T5 specimen, the *Q* values of 102.88–117.96 kJ/mol are in the range of 92 to 135 kJ/mol, i.e. between the activation energies for pipe diffusion and lattice self-diffusion of magnesium [29]. Kabirian and Mahmudi [30] reported that the *n* and *Q* values were 4.2–6.0 and 94.0–122.9 kJ/mol, respectively, for cast AZ91 alloy at 152–297 °C under constant punching stresses of 100–650 MPa, suggesting the activation of two parallel creep mechanisms of lattice and pipe-diffusion-controlled dislocation climb. The similar results are reported by Mahmudi et al. [15] and Nami et al. [31]. Therefore, dislocation climb controlled by pipe and lattice diffusion is the dominant creep mechanism for T5 specimen.

### 4.3. Effects of T5 Treatment on Creep Behavior

Generally, typical primary creep stage of the cast and aged Mg-Al-based alloys is characterized by continuously decelerating creep rate [13,14,15,16,17,24,25]. By contrast, HRRed AZ80-Ag alloy in the present work exhibits atypical primary creep stage at 150–175 °C (Figure 5). In general, the initial deceleration creep stage is related to the work hardening caused by dislocation pile-up. The increase of dislocation density near grain boundaries can provide preferential sites for the nucleation of DP, as shown in Figure 6b. It is reported that the growth of DP into grains develops with grain boundary sliding and migration [15,32]. Therefore, during initial creep process, GBS could be activated accompanying the nucleation and growth of DP. When the softening effect, derived from GBS and dynamic recovery, becomes predominant, a transition from deceleration to acceleration creep stage is achieved. With the full development of dynamic precipitation, GBS is reduced. Meanwhile, dynamic precipitates are obstacles to dislocation motion, improving work hardening effect. Thus, creep process gradually proceeds to the steady creep stage, where work hardening and softening effects reach a balance [33]. The atypical primary creep stage disappears at 120 °C, as few discontinuous precipitates are formed (Figure 6a).

Dynamic precipitation also occurs in the cast Mg-Al-based alloys during creep at 150–180 °C [13,15,16]. Generally, the volume fractions of dynamic discontinuous precipitates are relatively low [15,16], compared with that in the present work. Such difference might be mainly derived from the different initial microstructures between cast and wrought alloys. So it is inferred that the contribution of GBS to softening effect is limited during the creep of cast alloys, causing the absence of such atypical primary creep stage.

As for T5 specimen, since initial microstructure is already occupied by DP and CP, the growth of DP and resulting GBS are limited during creep process, which is inadequate for significant enhancement of softening effect. Consequently, the atypical primary creep stage is eliminated by aging treatment.

It is found that T5 treatment exerts a detrimental effect on the creep resistance. The microstructure of T5 specimen is mainly occupied by lamellar DP, which can provide more interfaces for GBS, making considerable contribution to creep deformation [34]. Besides, initial aging precipitates are prone to coarsen with increasing creep time, as demonstrated in Figure 8. Thus, their inhibition effect on dislocation motion is gradually weakened. As for HRRed specimen, the dynamic precipitation is quite limited at 120 °C. A high concentration of Al atoms in magnesium matrix is retained during creep, resulting in a strong solution hardening effect. Additionally, the contribution of GBS to creep is significantly reduced. At 150–175 °C, dynamic precipitation prevails during creep process. The volume fraction of DP in the crept HRRed specimen is smaller than that in T5 specimen, thus lower frequency of GBS may be activated in HRRed specimen. Moreover, less DP means more CP. Due to the smaller size and denser distribution, CP is more effective in hindering dislocation motion and GBS. As a result, HRRed specimen exhibits superior creep resistance to T5 specimen.

## 5. Conclusions

In the present study, effects of T5 treatment on microstructure and mechanical properties at elevated temperature of HRRed AZ80-Ag alloy were analyzed. The dynamic microstructural evolution and creep mechanism were also discussed. Main conclusions are drawn as follows:

(1) After T5 treatment, the microstructure is mostly occupied by discontinuous and continuous precipitates, with the former taking up a dominant volume fraction of ~64.9%. Besides, T5 treatment has little influence on the macro-texture.
(2) T5 treatment can improve the tensile strength at ambient temperature of the alloy, but deteriorate the tensile strength and creep resistance at elevated temperatures (120–175 °C), indicating opposite effects of T5 treatment on mechanical properties at ambient and elevated temperatures.
(3) During creep under conditions of 120–175 °C and 70–90 MPa, HRRed specimens exhibit accelerated dynamic precipitation behavior with increasing temperature. At 150–175 °C, the massive nucleation and growth of dynamic discontinuous precipitates could promote softening effect and lead to an atypical primary creep stage, which is eliminated by T5 treatment.
(4) Based on the creep stress exponent (2.97–6.23) and activation energy (102.88–141.99 kJ/mol), diffusion-controlled dislocation creep is the dominant creep mechanism for both HRRed and T5 specimens. Dislocations with <a> and <c> components are activated simultaneously, influencing creep behavior.

## Figures and Tables

**Table 1:** Tensile properties of HRRed and T5 specimens tested at different temperatures (standard deviation is given in parentheses).

Temperature	Condition	Yield Strength (MPa)	Ultimate Tensile Strength (MPa)	Elongation (%)
25 °C	HRRed	235.2 (7.5)	317.1 (0.9)	7.5 (0.4)
	T5	276.8 (1.8)	364.4 (3.1)	4.5 (0.5)
120 °C	HRRed	207.5 (5.5)	267.7 (7.9)	23.8 (1.0)
	T5	197.5 (3.3)	258.4 (0.5)	21.8 (0.8)
175 °C	HRRed	161.2 (11.0)	183.3 (7.8)	29.3 (5.2)
	T5	140.1 (8.5)	166.7 (6.8)	33.7 (3.9)

**Table 2:** Creep data of HRRed specimens tested at 120–175 °C and under 70–90 MPa.

Temperature (°C)	Stress (MPa)	Creep Life (h)	Strain (%)	Steady Creep Rate (s^−1^)
120	70	>100	0.27	1.50 × 10^−9^
	80	>100	0.46	2.45 × 10^−9^
	90	>100	0.52	3.15 × 10^−9^
150	70	>100	0.87	2.00 × 10^−8^
	80	>100	1.83	4.48 × 10^−8^
	90	>100	2.15	6.30 × 10^−8^
175	70	99.30	23.41	1.97 × 10^−7^
	80	51.26	24.73	4.01 × 10^−7^
	90	34.11	24.28	6.60 × 10^−7^

**Table 3:** Creep data of T5 specimens tested at 120–175 °C and under 70–90 MPa.

Temperature (°C)	Stress (MPa)	Creep Life (h)	Strain (%)	Steady Creep Rate (s^−1^)
120	70	>100	0.57	4.86 × 10^−9^
	80	>100	0.83	7.29 × 10^−9^
	90	>100	1.06	1.32 × 10^−8^
150	70	>100	2.21	5.52 × 10^−8^
	80	>100	6.45	1.30 × 10^−7^
	90	>100	13.57	2.20 × 10^−7^
175	70	76.14	18.76	2.27 × 10^−7^
	80	29.69	20.65	5.83 × 10^−7^
	90	16.31	21.61	1.08 × 10^−6^
